# Anticoagulation strategy and safety in critically ill COVID-19 patients: a French retrospective multicentre study

**DOI:** 10.1186/s12959-023-00491-6

**Published:** 2023-04-18

**Authors:** Pauline Lamouche-Wilquin, Léa Perrin, Morgane Pere, Matthieu Raymond, Pierre Asfar, Cedric Darreau, Florian Reizine, Gwenhaël Colin, Agathe Delbove, Johann Auchabie, Baptiste Hourmant, Aurélien Frérou, Béatrice La Combe, Jean Morin, Pierre Kergoat, Julien Lorber, Pierre-Yves Egreteau, Jérome Souchard, Emmanuel Canet, Jean-Baptiste Lascarrou

**Affiliations:** 1 Service de Médecine Intensive Réanimation, Centre Hospitalier Universitaire de Nantes, 30 Bd Jean Monet, Nantes Cedex 9, 44093 France; 2 Service de Médecine Intensive Réanimation, Centre Hospitalier de Saint-Nazaire, Saint-Nazaire, France; 3 Plateforme de Méthodologie et Biostatistique, Centre Hospitalier Universitaire de Nantes, Nantes, France; 4 Service de Médecine Intensive Réanimation, Centre Hospitalier Universitaire d’Angers, Angers, France; 5 Service de Médecine Intensive Réanimation, Centre Hospitalier Le Mans, Angers, France; 6 Service de Médecine Intensive Réanimation, Centre Hospitalier Universitaire de Rennes, Rennes, France; 7 Service de Médecine Intensive Réanimation, Centre Hospitalier Départemental de Vendée, La Roche-sur-Yon, France; 8 Service de Réanimation Polyvalente, Centre Hospitalier Bretagne Atlantique, Vannes, France; 9 Service de Réanimation Polyvalente, Centre Hospitalier de Cholet, Cholet, France; 10 Service de Médecine Intensive Réanimation, Centre Hospitalier Universitaire de Brest, Brest, France; 11 Service de Réanimation Polyvalente, Centre Hospitalier de Saint-Malo, Saint-Malo, France; 12 Service de Réanimation Polyvalente, Centre Hospitalier Bretagne Sud, Lorient, France; 13 Service de Soins Intensifs de Pneumologie, Centre Hospitalier Universitaire de Nantes, Nantes, France; 14 Service de Réanimation Polyvalente, Centre Hospitalier de Cornouaille, Quimper, France; 15 Service de Réanimation Polyvalente, Centre Hospitalier de Morlaix, Morlaix, France; 16 Service de Réanimation Chirurgicale, Centre Hospitalier Universitaire de Rennes, Rennes, France

**Keywords:** COVID-19, Anticoagulation strategies, Venous thromboembolism, Deep vein thrombosis, Pulmonary embolism, Intensive care unit

## Abstract

**Background:**

Patients with critical illness due to COVID-19 exhibit increased coagulability associated with a high risk of venous thrombo-embolism (VTE). Data on prophylactic anticoagulation for these patients are limited and conflicting. The purpose of this study was to evaluate whether intermediate-dose prophylactic anticoagulation in patients with COVID-19 requiring ICU admission was associated with better outcomes compared to standard-dose prophylactic anticoagulation.

**Methods:**

We retrospectively included adults admitted with severe COVID-19 to any of 15 ICUs, in 2020 or 2021. We compared the groups given intermediate-dose vs. standard-dose prophylactic anticoagulation. The primary outcome was all-cause day-90 mortality. Secondary outcomes were VTE (pulmonary embolism or deep vein thrombosis), ICU stay length, and adverse effects of anticoagulation.

**Results:**

Of 1174 included patients (mean age, 63 years), 399 received standard-dose and 775 intermediate-dose prophylactic anticoagulation. Of the 211 patients who died within 90 days, 86 (21%) received intermediate and 125 (16%) standard doses. After adjustment on early corticosteroid therapy and critical illness severity, there were no significant between-group differences in day-90 mortality (hazard ratio [HR], 0.73; 95%CI, 0.52–1.04; *p* = 0.09) or ICU stay length (HR, 0.93; 95%CI, 0.79–1.10; *p* = 0.38). Intermediate-dose anticoagulation was significantly associated with fewer VTE events (HR, 0.55; 95%CI, 0.38–0.80; *p* < 0.001). Bleeding events occurred in similar proportions of patients in the two groups (odds ratio, 0.86; 95%CI, 0.50–1.47; *p* = 0.57).

**Conclusions:**

Mortality on day 90 did not differ between the groups given standard-dose and intermediate-dose prophylactic anticoagulation, despite a higher incidence of VTE in the standard-dose group.

**Supplementary Information:**

The online version contains supplementary material available at 10.1186/s12959-023-00491-6.

## Introduction

The inflammatory response to COVID-19 triggers coagulation disorders that can cause venous thrombo-embolism (VTE) [1–5]. Autopsy studies have evidenced high frequencies of both thrombotic microangiopathy, notably in the lungs, and macrovascular thrombotic events such as deep vein thrombosis (DVT) and pulmonary embolism (PE) [6, 7]. Moreover, a high incidence of clinical PE was reported early in the pandemic [8, 9]. In patients with acute respiratory distress syndrome (ARDS), the cumulative incidence of PE was 11.7% in patients with COVID-19 and 2.1% in those with other diagnoses [8, 10]. Interestingly, PE was not always associated with DVT, suggesting in situ thrombosis at sites of microangiopathy rather than migration of emboli [11]. The complex process of runaway inflammation that occurs during severe COVID-19 may contribute to vascular obstruction by inducing capillary damage, thrombosis, and even organ dysfunction [12, 13]. The high incidences of DVT and PE in patients with COVID-19 have prompted some experts to advocate higher-than-standard prophylactic anticoagulation dosages in patients with risk factors [14]. Others, however, continue to recommend standard dosages [15]. These differences of opinion may be ascribable to the lack of convincing evidence that intensified anticoagulation improves survival or decreases the duration of invasive mechanical ventilation [16]. Several observational studies in small populations demonstrated a significant reduction in VTE with intermediate- or therapeutic-dose anticoagulant regimens used for prevention [7, 13–15, 17], although mortality was unchanged. The REMAP-CAP randomised controlled trial of therapeutic-dose versus standard-dose prophylactic anticoagulation in patients admitted to the intensive care unit (ICU) for severe COVID-19 was stopped early because the therapeutic dosage failed to decrease the number of days without organ failures [18]. This result would not seem to support routine therapeutic-dose anticoagulation in ICU patients with COVID-19. However, D-dimer elevation at admission has been reported to be associated with greater severity and higher mortality in patients with COVID-19 [19]. Consequently, higher anticoagulant doses might be warranted in patients with early D-dimer elevation. Thus, the current evidence fails to conclusively indicate whether and when standard- or intermediate-dose prophylactic anticoagulation is optimal.

The aim of this retrospective multicentre study was to compare standard-dose vs. intermediate-dose anticoagulation used to prevent VTE in ICU patients with COVID-19-related acute respiratory failure. The primary outcome was mortality 90 days after ICU admission. Given the non-randomised design of our study, we adjusted the statistical analyses on the main known determinant of ICU mortality, namely, the SAPS II score [20], and on early corticosteroid therapy defined as started before or within 24 h after ICU admission [21].

## Methods

### Study design and participants

We retrospectively collected data for patients admitted to any of 15 ICUs in western France between 1 and 2020 and 31 December 2021. The data for each patient were entered into an electronic case-report form (Castor EDC, Amsterdam, The Netherlands) by the investigators in each participating centre.

Patients older than 18 years were eligible for inclusion if they required ICU admission due to severe lung disease with a positive reverse transcriptase-polymerase chain reaction test for SARS-CoV-2 in one or more upper and/or lower respiratory tract samples. Non-inclusion criteria were pregnancy, guardianship, therapeutic-dose anticoagulation for comorbidities or prevention of COVID-19-related VTE, and PE diagnosed by computed tomography pulmonary angiography (CTPA) before or within 24 h after ICU admission.

### Definitions

We defined standard-dose prophylactic anticoagulation as subcutaneous low-molecular-weight heparin (usually enoxaparin in a dosage of 4000 IU/day or low-dose unfractionated heparin, usually calciparine, in a dosage of 500 IU/kg/day in two or three injections depending on body weight) [22]. We defined intermediate-dose prophylactic anticoagulation as subcutaneous low-molecular-weight heparin in a dosage of about 1 mg/kg/day [23], e.g., 6000 IU/24 h for patients with a body mass index (BMI) below < 30 kg/m^2^ or 4000 IU/12 hours for those with a BMI > 30 kg/m^2^.

The anticoagulation regimen prescribed at ICU admission followed the local protocol in each participating ICU. This regimen was kept unchanged unless thrombosis occurred. No changes in local protocols occurred during the study enrolment period.

In patients who had acute or chronic renal failure with a creatinine clearance below 30 mL/min/1.73 m^2^, intermediate-dose prophylactic anticoagulation was defined as unfractionated heparin with an anti-factor Xa assay target of 0.2 to 0.3 IU/mL [24].

DVT was defined as complete obstruction of a deep vein in an upper or lower limb by a thrombus formed in situ, confirmed by Doppler ultrasound [25]. Doppler ultrasound was performed when DVT was suspected during daily clinical screening by the bedside intensivist. PE was defined as complete or partial obstruction of a pulmonary main artery or branch, confirmed by CTPA. CTPA was performed routinely before or within 24 h after ICU admission.

Major bleeding was defined as type ≥ 3 bleeding according to the Bleeding Academic Research Consortium (BARC) scale [26]. Type 3 bleeding includes (a) overt bleeding responsible for a haemoglobin drop of 3–5 g/dL or leading to blood transfusion; (b) overt bleeding responsible for a haemoglobin drop of 5 g/dL or cardiac tamponade or need for surgical haemostasis or need for intravenous vasoactive drugs; and (c) intracranial or intraspinal bleeding or intraocular bleeding compromising vision. Type 4 is coronary-artery bypass grafting-related bleeding or perioperative intracranial bleeding within 48 h or need for surgical haemostasis after sternotomy closure or transfusion of 5 units of whole blood or packed red blood cells within a 48-hour period or chest-tube output of at least 2 L within a 24-hour period. Finally, type 5 includes (a) death probably due to bleeding but without imaging or autopsy confirmation and (b) fatal bleeding, either overt or confirmed by imaging or autopsy.

### Outcomes

The primary outcome was mortality on day 90. We compared the primary outcome in the groups with intermediate-dose vs. standard-dose prophylactic anticoagulation. Secondary outcomes were ICU stay length, proportion of patients with VTE, and proportions of patients with major bleeding and with blood transfusion.

### Data collection

At each centre, the study investigator used standardised forms to collect the data listed in Table 1; presence of co-infection; and use of antiviral agents, immunomodulatory drugs, and/or initial antibiotics.

**Table 1:** Demographics and clinical features at ICU admission and during the ICU stay of patients with severe COVID-19 given standard-dose or intermediate-dose prophylactic anticoagulation

	Total N = 1174	Intermediate N = 775	Standard N = 399	*p* value
Baseline characteristics				
Age, y, mean (SD)	62 (13)	63 (12)	61 (13)	0.082
Males, n (%)	805 (68)	525 (68)	280 (70)	0.39
BMI, kg/m^2^, mean (SD)	29 (6)	30 (6)	28 (6)	0.0002
Hypertension, n (%)	604 (51)	410 (52)	194 (48)	0.16
Current smoker, n (%)	71 (6)	43 (5)	28 (7)	0.29
Diabetes, n (%)	338 (28)	217 (28)	121 (30)	0.40
Immunosuppression, n (%)	161 (13)	103 (13)	58 (14)	0.55
Charlson Comorbidity Index^a^, mean (SD)	3.43 (2.53)	3.55 (2.54)	3.20 (2.49)	0.02
Hospital stay ≥ 48 h within past 3 months, n (%)	84 (7)	51 (6)	33 (8)	0.28
**COVID-19 data**				
Vaccination, n (%)	49 (15)	46 (16)	3 (5)	0.03
Symptom onset to ICU admission, days, mean (SD)	8.06 (11.93)	7.83 (14.30)	8.51 (4.50)	0.82
Variants, n (%)				
Historical	852 (72)	530 (68)	322 (80)	
Alpha	85 (7)	80 (10)	5 (1)	
Beta	14 (1)	14 (2)	0 (0)	
Delta	118 (10)	108 (14)	10 (2)	
Omicron	1 (0.08)	1 (0.1)	0 ()	
Unknown	104 (9)	42 (5)	62 (15)	
**Features at ICU admission**				
Respiratory rate, breaths/min, mean (SD)	26.08 (6.27)	25.75 (6.29)	26.71 (6.18)	0.01
Respiratory support on ICU day 1, n (%)				
Standard oxygen therapy	683 (59)	405 (53)	278 (70)	
HFNO	337 (29)	278 (36)	59 (15)	< 0.0001
NIV	15 (1)	11 (1)	4 (1)	
iMV	125 (11)	71 (9)	54 (13)	
PaO_2_/FiO_2_, mean (SD)				
Standard oxygen therapy	185 (96)	167 (80.72)	201 (109.41)	< 0.001
HFNO or iMV	128 (61.64)	124 (61.17)	138 (61.98)	< 0.01
Lymphocytes mean (SD)	1.16 (4)	1.16 (4)	1.45 (5)	0.39
CRP, mg/L, mean (SD)	135.91 (91)	131.36 (90)	144.61 (93)	0.07
Corticosteroids, n (%)	850 (72)	684 (88)	166 (41)	< 0.001
IL6 antagonist therapy, n (%)	233 (19)	227 (29)	6 (1)	< 0.001
SOFA^b^ score, mean (SD)	3.94 (2.60)	3.71 (2.48)	4.37 (2.75)	< 0.0001
SAPS II score^c^, mean (SD)	32.81 (13.77)	32.20 (13.18)	33.96 (14.75)	0.04
**Treatments in the ICU**				
Mechanical ventilation, n (%)	665 (66)	419 (59)	246 (81)	< 0.001
Days on mechanical ventilation, mean (SD)	20.33 (19)	21.08 (20)	19.04 (16)	0.53
Neuromuscular blocking agents, n (%)	593 (50)	377 (49)	216 (54)	0.07
Prone positioning, n (%)	455 (68)	296 (70)	159 (64)	0.81
VV-ECMO, n (%)	47 (7)	29 (6)	18 (7)	0.52
Vasopressor use, n (%)	484 (41)	295 (38)	189 (47)	< 0.01
Renal replacement therapy, n (%)	102 (8)	53 (6)	49 (12)	0.07

^a^[20]

^b^[22], determined 24 h after ICU admission

^c^[23], determined 24 h after ICU admission

BMI: body mass index; ICU: intensive care unit; HFNO: high-flow nasal oxygen therapy; NIV: non-invasive ventilation; iMV: invasive mechanical ventilation; CRP: C-reactive protein; SOFA: Sequential Organ Failure Assessment; SAPS II: Simplified Acute Physiology Score version II; PaO_2_: arterial partial pressure of oxygen; FiO_2_: fraction of inspired oxygen; VV-ECMO: veno-venous extra-corporeal membrane oxygenation

### Statistical analysis

Categorical variables were described as count (percentage) and continuous variables as mean ± SD if normally distributed and as median [interquartile range] otherwise. Normality was assessed by visual inspection of the distribution curve. For comparisons of the intermediate-dose and standard-dose groups, we applied the chi-square test or Fisher’s exact test for categorical variables and Student’s *t*-test or the Mann-Whitney U test for quantitative variables, as appropriate.

Day-90 mortality was compared between groups by building a Cox model adjusted for the SAPS II score [21] for early corticosteroid therapy defined as started before or within 24 h after ICU admission [27]. Centre was included as a random effect. A Kaplan-Meier plot was also produced. ICU stay length and the proportion of patients with VTE were compared between groups using a Fine-and-Gray competitive-risk model with death as the competing event and adjustment for the SAPS II score and early corticosteroid therapy [28]. Finally, we compared the sub-groups of patients with D-dimer levels no higher than 1000 ng/mL vs. higher than 1000 ng/mL at ICU admission; when the D-dimer level was unavailable, the dichotomising criterion was a fibrinogen level no higher than vs. higher than 4 g/L [20, 29].

The statistical analysis was performed using SAS software version 9.4 (SAS Institute, Cary, NC). All tests were two-sided, and *p* values smaller than 0.05 were considered significant.

## Results

### Patient characteristics

Between 1 and 2020 and 31 December 2021, 1449 patients with COVID-19 were admitted to the 15 participating ICUs. After exclusion of the 275 patients who received therapeutic-dose anticoagulation (for VTE or as required per prophylaxis by local protocol), 1174 patients were left for the analysis, including 775 in the intermediate group and 399 in the standard group (eFigure 1).

Table 1 reports the main characteristics of the patients. Disease severity assessed using the SOFA score and SAPS II was greater in the standard group. Both early corticosteroid therapy and interleukin-6-antagonist therapy were used significantly more often in the intermediate than in the standard patients. In the standard group, median time to corticosteroid initiation was 0 [-1 to 0] days, mean duration was 9.0 ± 4.5 days, and mean dosage was 80 ± 156 mg/day prednisone-equivalent, with dexamethasone used in 75% of patients; corresponding values in the intermediate group were 0 [-2 to 0] days, 9 ± 14 days, and 48 ± 31 mg/day prednisone-equivalent, with dexamethasone used in 95% of patients.

### Day-90 mortality (primary outcome measure)

**Fig. 1:**
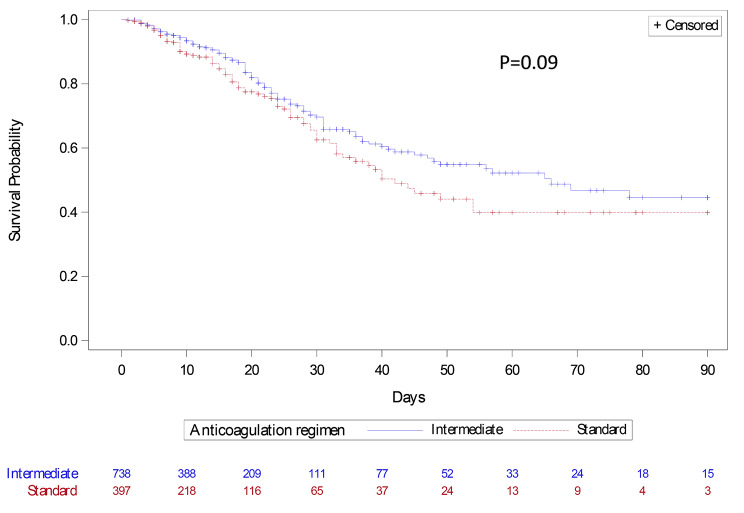
Kaplan-Meier plot of day-90 survival

Table 2 reports the main outcomes of the patients. Of the 1174 patients, 211 died within 90 days, 86 (21%) in the standard group and 125 (16%) in the intermediate group (-5.4; 95% confidence interval [95%CI], -10.2 to -0.6). The Cox model comparing day-90 survival after adjustment for the SAPS II score and early corticosteroid therapy showed no significant difference between the intermediate and standard groups (hazard ratio [HR], 0.73; 95%CI, 0.52–1.04; *p* = 0.09) (Fig. 1).

**Table 2:** Outcomes in the intermediate-dose and standard-dose prophylactic anticoagulation groups

	Total N = 1174	Intermediate N = 775	Standard N = 399	Crude difference (95%CI)	*p* value
**Vital status on day 90**					
Dead, n (%)	211 (18)	125 (16)	86 (21)	-5.4 (-10.2 to -0.6)	-
Alive, out of the ICU, n (%)	945 (80)	634 (82)	311 (78)	4.0 (-0.9 to 8.9)	
Alive, still in the ICU, n (%)	17 (1)	15 (2)	2 (0.5)	1.4 (0.2 to 2.6)	
**Secondary endpoints**					
ICU stay length, days, mean (SD)	16 (18)	16 (19)	15.84 (17)	1.1 (-1.2 to 3.3)	-
DVT, n (%)	107 (9)	59 (7)	48 (12)	-4.4 (-8.1 to -0.7)	0.013
PE, n (%)	108 (9)	55 (7)	53 (13)	-6.2 (-10.0 to -2.4)	0.0005

DVT: deep vein thrombosis; PE: pulmonary embolism; 95%CI: 95% confidence interval

When we compared patients with D-dimer levels no greater than vs. greater than 1000 ng/mL (or fibrinogen no greater than vs. greater than 4 g/L), we also found no significant difference in day-90 mortality (HR, 0.80; 95%CI, 0.55–1.17; *p* = 0.25) (eFigure 2).

### ICU stay length

Overall, length of stay in the ICU was 10 [4.00–22.00] days. The Fine-and-Gray competitive risk model, with death as a competing event and adjustment for the SAPS II score and early corticosteroid therapy, showed no significant difference between the two groups regarding ICU stay length (HR, 0.93; 95%CI, 0.79–1.10; *p* = 0.38) (eFigure 3).

### Frequency of venous thrombo-embolism

Of the 1174 patients, 186 (16%) experienced at least one VTE event. Details are given in eTable1. Mean time from ICU admission to VTE diagnosis was 11 ± 9 days.

PE was diagnosed in 111 (9%) patients overall, 58 (7%) in the intermediate group and 53 (13%) in the standard group. Segmental arteries were predominantly involved (n = 60, 54%), followed by sub-segmental arteries (n = 31, 28%) then proximal arteries (n = 19, 17%). Mean time from ICU admission to PE diagnosis was 9 ± 7 days overall. PE was significantly more common in the standard than in the intermediate patients (*p* = 0.0005).

DVT was diagnosed in 107 (9%) patients overall, 54 (7%) in the intermediate group and 48 (12%) in the standard group. Mean time from ICU admission to DVT diagnosis was 16 ± 14 days in the intermediate and 14 ± 8 days in the standard patients. DVT was significantly more common in the standard than in the intermediate patients (*p* = 0.013).

**Fig. 2:**
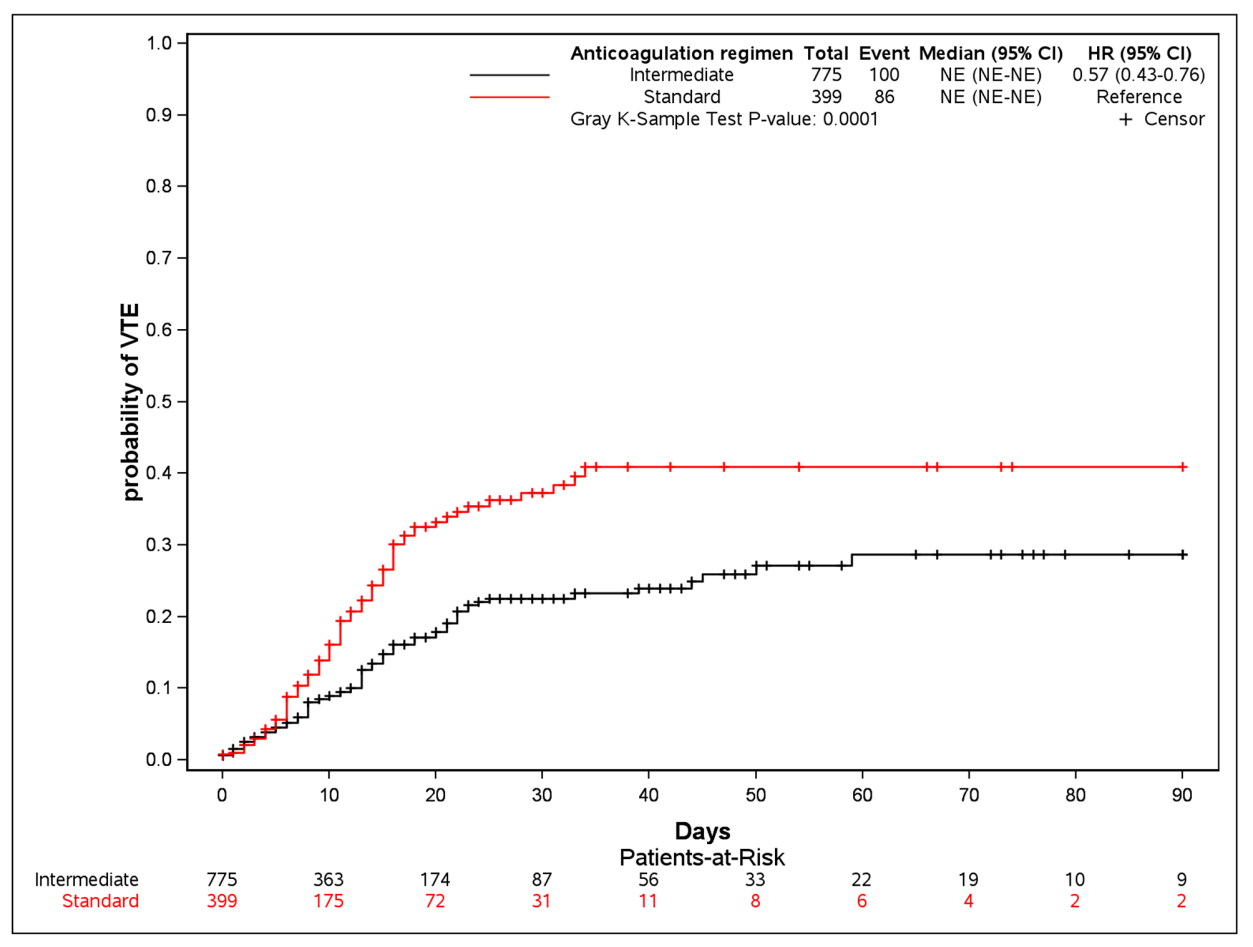
Cumulative incidence of venous thromboembolism with death as a competing event

In the multivariable analysis adjusted for the SAPS II score and early corticosteroid therapy, intermediate-dose prophylactic anticoagulation was significantly associated with a lower frequency of VTE (HR, 0.55; 95%CI, 0.38–0.80; *p* = 0.0018) (Fig. 2).

### Adverse events

Major bleeding occurred in 95 (8%) patients and blood transfusion was required in 163 (14%) patients (Table 3). By logistic regression adjusted for the SAPS II score and early corticosteroid therapy, the frequency of major bleeding and/or blood transfusion was not significantly different between the two groups (odds ratio, 0.86; 95%CI, 0.50–1.47; *p* = 0.57).

**Table 3:** Adverse events potentially related to anticoagulation

	Total N = 1174	Intermediate anticoagulation N = 775	Standard anticoagulation N = 399	Crude difference (95%CI)	*p* value
**Bleeding**, n (%)	95 (8)	61 (8)	34 (8)	-0.6 (-4.0 to 2.7)	0.57
Gastro-intestinal tract, n (%)	36 (4)	25 (3)	11 (3)	0.5 (-1.6 to 2.5)	0.66
Intracranial, n (%)	10 (1)	5 (1)	5 (1)	-0.6 (-1.8 to 0.6)	0.32
Deep haematoma, n (%)	8 (1)	7 (1)	1 (0.25)	0.7 (-0.2 to 1.5)	0.28
Blood transfusion*, n (%)	163 (14)	98 (12.7)	65 (16.3)	-3.7 (-8.0 to 0.6)	0.08

*Patients given blood transfusions either had major bleeding (n = 95) or had low red-cell counts without evidence of active bleeding recorded in the medical files (n = 68)

## Discussion

In this large retrospective multicentre study, day-90 mortality was not different between groups given standard-dose vs. intermediate-dose prophylactic anticoagulation. PE and DVT each occurred in 9% of the patients overall, and both were significantly more common with the standard dose; 29 patients (2.5%) with DVT also had PE. Major bleeding was experienced by similar proportions of patients in the two groups.

VTE occurred in 16% of our patients. In a retrospective study of 184 ICU patients given at least standard-dose prophylactic anticoagulation, PE was the predominant thrombo-embolic event and was more common than in our study (14% vs. 9% overall), whereas DVT was less common (1.6% vs. 9%) [30]. In a far larger study of 3334 hospitalised patients, most of whom received standard-dose prophylactic anticoagulation, the incidences were only 3.2% for PE and 3.9% for DVT [31]. However, only a fourth of the patients required ICU admission, and in this sub-group the incidence of PE and/or DVT was 13.6%. Heterogeneity in healthcare systems, notably in the algorithms used to diagnose PE and DVT, may affect the anticoagulation strategy at the centre level [32].

In a meta-analysis with over 18 000 patients admitted to a ward or ICU for COVID-19, any anticoagulation (therapeutic, intermediate, or prophylactic) was associated with a 50% reduction in all-cause hospital mortality (relative risk, 0.50; 95% confidence interval, 0.40–0.62) [33]. The decrease in mortality was even higher in patients who required ICU admission and received therapeutic-dose anticoagulation (0.30; 0.15–0.60). However, therapeutic doses were associated with a higher risk of bleeding. Another meta-analysis, however, which had data for 5700 patients, compared standard-dose to either intermediate- or therapeutic-dose prophylactic anticoagulation and found no significant difference in hospital mortality; with the higher doses, thrombo-embolic events were less common and bleeding more common [34]. Moreover, large, well-done, randomised, controlled trials (ATTACC, ACTIV-4a, and REMAP-CAP [19]) failed to demonstrate any benefits of therapeutic-dose anticoagulation on mortality or organ dysfunction. Thus, the evidence does not support therapeutic-dose anticoagulation for prophylactic purposes in patients with critical illness due to COVID-19. The randomised controlled trial INSPIRATION in 562 ICU patients focussed on intermediate-dose prophylactic anticoagulation [35]. Compared to standard-dose prophylactic anticoagulation, the frequency of the composite primary outcome comprising 30-day mortality, venous and arterial thrombosis, and extracorporeal membrane oxygenation was not significantly different, and neither did bleeding differ in frequency, although severe thrombocytopenia occurred only in the intermediate-dose group [35]. This study cannot be readily compared to ours, however, given the difference in the primary outcome. A smaller randomised controlled trial of intermediate- vs. standard-dose prophylactic anticoagulation included 176 patients who either required ICU admission or had coagulopathy [36]. There were no differences in the primary outcome of 30-day mortality or the secondary outcomes of arteriovenous thrombosis and bleeding. A retrospective observational study compared standard thromboprophylaxis to enhanced thromboprophylaxis, which usually consisted in a higher-than-intermediate dose of 100 to 200 IU/kg/day of enoxaparin [37]. By propensity-matched analysis, ICU mortality was significantly lower with the enhanced regimen. However, the absence of a between-group difference in the frequency of thrombo-embolic events suggests potential unrecognised selection bias in this non-randomised study [37]. Finally, a retrospective review of 565 ICU patients with propensity-score matching compared standard- to intermediate-dose enoxaparin for prophylaxis [38]. The two groups were not significantly different for 30-day mortality, hospital mortality, VTE, or any thrombo-embolism. Thus, the overall body of data would not seem to support higher-than-standard doses of prophylactic anticoagulation in critically ill COVID-19 patients.

The effect of anticoagulation may vary with time of initiation, degree of coagulation activation, and severity of inflammation [39–41]. D-dimer elevation was associated with severe disease and death among patients with COVID-19 [42, 43]. In a meta-analysis, D-dimer levels above the upper limit of normal were significantly associated with both severe disease (relative risk [RR], 1.58; 95%CI, 1.25–2.00; *p* < 0.0001) and mortality (RR, 1.82; 95%CI, 1.40–2.37; *p* < 0.0001) [44]. In our overall population, two-thirds of patients had high D-dimer or fibrinogen levels. Despite this high prevalence, VTE occurred in only 16% of patients. Thus, the clinical relevance of high D-dimer and fibrinogen levels is unclear [45], perhaps due in part to variations in assays and in the cut-offs used to define normal ranges [46]. Importantly, day-90 mortality was not higher in patients with D-dimer or fibrinogen elevation compared to those with normal values for these parameters.

CTPA is now widely performed to detect PE in patients admitted for severe COVID-19. Data suggesting that CTPA may lead to PE overdiagnosis were reported several years before the COVID-19 pandemic [47]. In our cohort, 28% of PE cases involved sub-segmental arteries. Recent guidelines suggest withholding anticoagulation in patients with sub-segmental PE who have no risk factors for thrombosis recurrence, no evidence of DVT on serial imaging, and good cardiorespiratory reserve [48]. However, these criteria are unlikely to be met by patients with critical illness due to COVID-19. More research is needed to determine the optimal anticoagulation strategies for preventing and treating PE in patients with COVID-19.

The retrospective design is a major limitation of our study. Another is that our patients were managed over the first two years of the pandemic, during which the treatment of severe COVID-19 underwent considerable changes driven by robust scientific evidence. In particular, routine dexamethasone was introduced in July 2020 [41]. Disease severity was greater in our standard group, which received a higher mean corticosteroid dose. Dexamethasone decreases lung inflammation, thereby potentially diminishing the risk of PE. We adjusted our analysis for the use of early corticosteroid therapy but not for corticosteroid dose, which may have resulted in bias. Third, CTPA was performed routinely at or within 24 h after ICU admission, whereas Doppler ultrasonography to detect DVT was done only when DVT was suspected clinically. Moreover, repeat CTPA to diagnose PE occurring during the ICU stay was also performed only based on a clinical suspicion. Thus, PE at ICU admission may have been overdiagnosed, while DVT and PE during the ICU stay may have been underdiagnosed. Finally, mortality was lower in our study than in earlier reports [49]. One possible explanation is the younger age of our patients (62 ± 13 years). Moreover, the inclusion period extended well into the pandemic, until the end of 2021, when the management of severe COVID-19 had improved [50]. Finally, all participating ICUs were in western France, where COVID-19 had a lower incidence than in other parts of the country and, therefore, put less strain on the healthcare system. This fairly low mortality may have limited our ability to detect a difference in mortality between our two study groups.

## Conclusion

Day-90 mortality did not differ between the groups given standard-dose vs. intermediate-dose prophylactic anticoagulation in the analysis adjusted for critical-illness severity and early corticosteroid therapy. However, VTE was more common in the standard-dose group. Our findings suggest that either standard-dose or intermediate-dose prophylactic anticoagulation can be used in patients with critical COVID-19 illness. Local practices might deserve to be adapted for specific sub-groups, which remain to be identified. The World Health Organisation is planning a meta-analysis that may provide information of relevance to clinical practice.

## Electronic supplementary material

Below is the link to the electronic supplementary material.

Supplementary Material 1

Supplementary Material 2

Supplementary Material 3

Supplementary Material 4

## Data Availability

The datasets used and analysed during the current study are available from the corresponding author on reasonable request.

## Abbreviations

95%CI: 95% confidence interval
ARDS: acute respiratory distress syndrome
COVID-19: coronavirus disease
CTPA: computed tomography pulmonary angiography
DVT: deep vein thrombosis
OR: odds ratio
PE: pulmonary embolism
SAPS II: Simplified Acute Physiology Score version II
SARS-CoV-2: severe acute respiratory syndrome coronavirus type 2
VTE: venous thromboembolism
