# Supplementary figures and images for: Anticoagulation strategy and safety in critically ill COVID-19 patients: a French retrospective multicentre study
Source: Thromb J. 2023 Apr 18;21:42. doi: 10.1186/s12959-023-00491-6 (PMC10112319; doi:10.1186/s12959-023-00491-6)

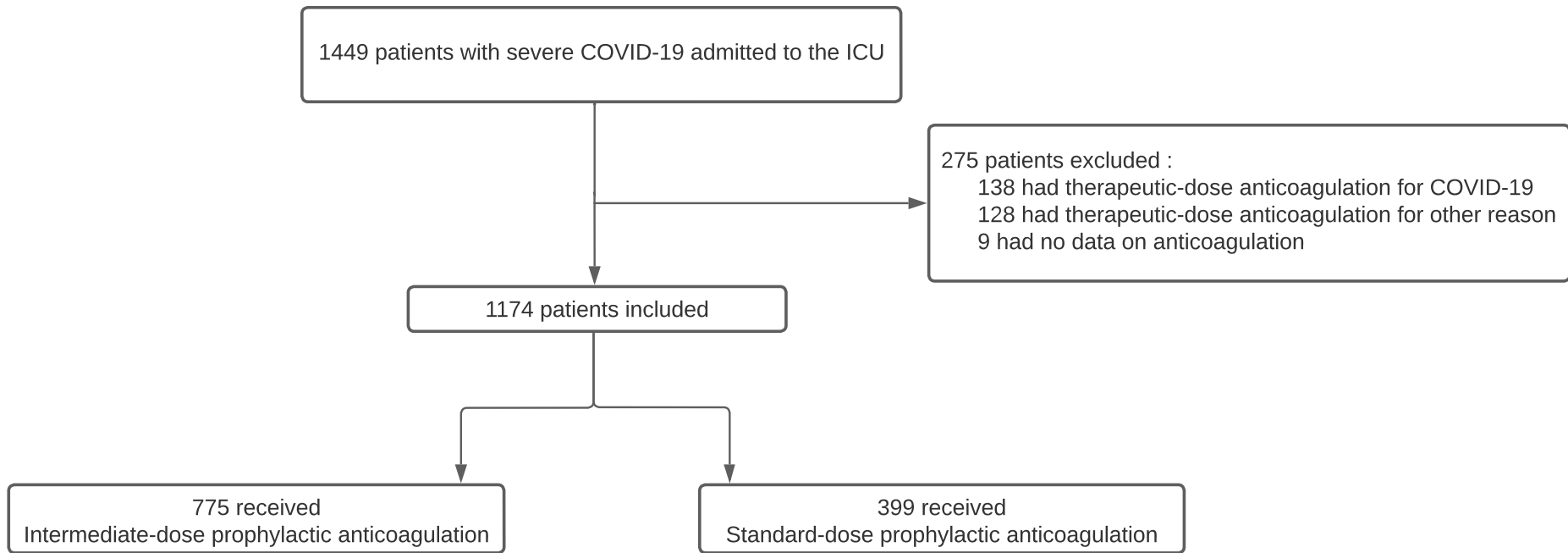

Supplement: Supplementary file 1 — Supplementary Material 1 [file 12959_2023_491_MOESM1_ESM.pdf]

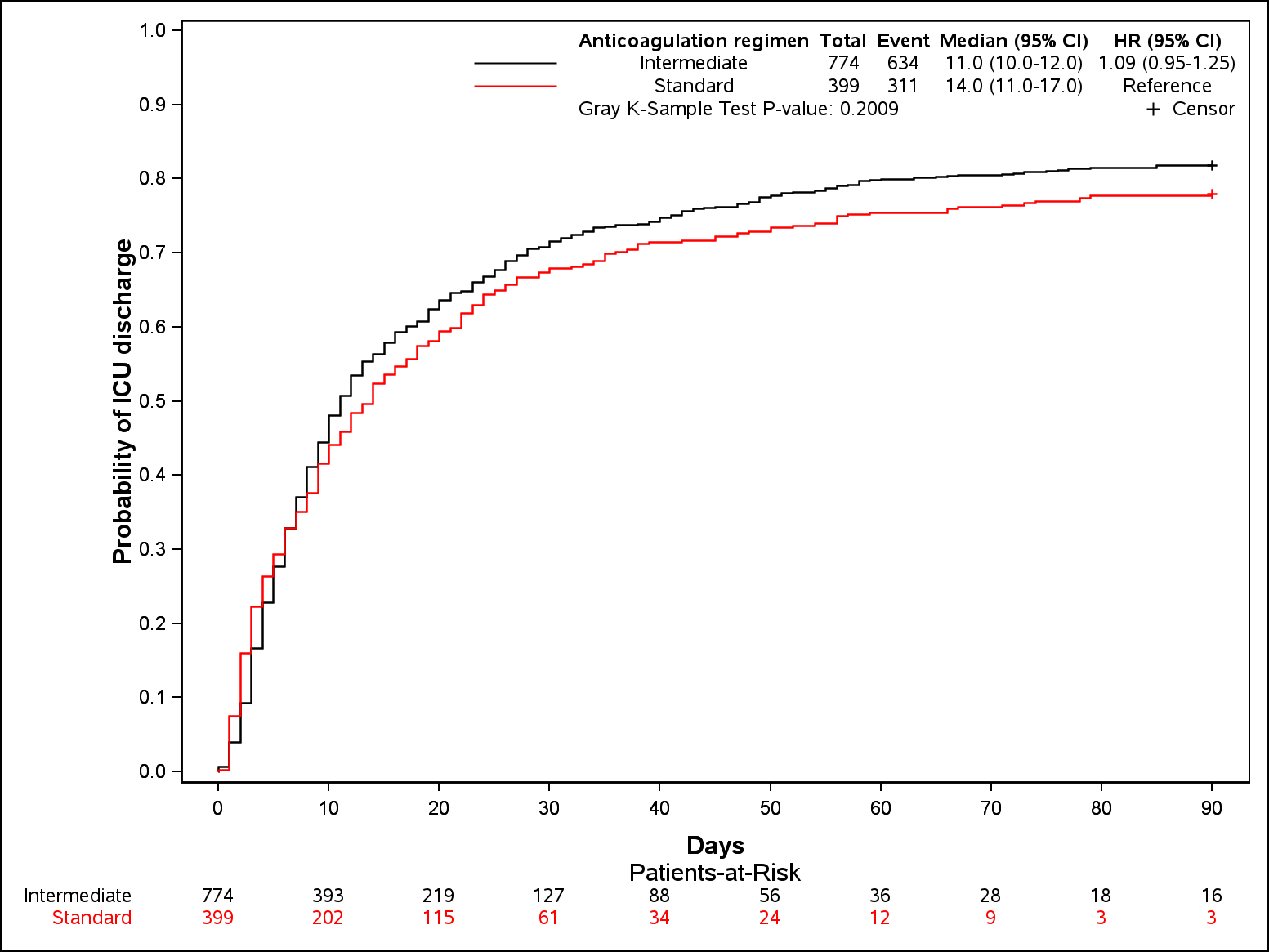
**eFigure 3. Cumulative incidence of ICU discharge with death as a competing event**

Supplement: Supplementary file 4 — Supplementary Material 4 [file 12959_2023_491_MOESM4_ESM.docx]
