# Supplementary material for: Anticoagulation strategy and safety in critically ill COVID-19 patients: a French retrospective multicentre study
Source: Thromb J. 2023 Apr 18;21:42. doi: 10.1186/s12959-023-00491-6 (PMC10112319; doi:10.1186/s12959-023-00491-6)
Supplement: Supplementary file 2 — Supplementary Material 2 [file 12959_2023_491_MOESM2_ESM.docx]

|  | **Total**  **N= 1174** | **Intermediate**  **N= 775** | **Standard**  **N= 399** |
| --- | --- | --- | --- |
| **PE, n (%)** | 111 (9.45) | 58 (7.48) | 53 (13.28) |
| Proximal | 19 (17.11) | 12 (20.68) | 7 (13.20) |
| Segmental | 60 (54.00) | 29 (50.00) | 32 (60.38) |
| Sub-segmental | 31 (27.92) | 17 (29.31) | 14 (26.42) |
| **DVT, n (%)** | 107 (9.12) | 59 (7.62) | 48 (12.03) |
| Lower limb | 76 (71.03) | 38 (64.41) | 38(79.17) |
| Upper limb | 6 (5.61) | 3 (5.08) | 3 (6.25) |
| Central or dialysis line | 18 (16.82) | 12 (20.34) | 6 (12.5) |
| ECMO cannula | 5 (4.67) | 4(6.78) | 1(2.08) |
| Other site | 2 (1.87) | 2 (3.39) | 0 (0) |

**eTable 1: Incidence and sites of venous thromboembolism**

PE: pulmonary embolism; DVT: deep vein thrombosis; ECMO: extra-corporeal membrane oxygenation
