# Supplementary material for: Anticoagulation strategy and safety in critically ill COVID-19 patients: a French retrospective multicentre study
Source: Thromb J. 2023 Apr 18;21:42. doi: 10.1186/s12959-023-00491-6 (PMC10112319; doi:10.1186/s12959-023-00491-6)
Supplement: Supplementary file 3 — Supplementary Material 3 [file 12959_2023_491_MOESM3_ESM.docx]

**eFigure 2. Kaplan-Meier plots of day-90 survival in the sub-groups with D-dimer levels no higher vs. higher than 1000 ng/mL.**

P=0.25

D-dimers > 1000 ng/mL (if unavailable, fibrinogen >4 g/L)

D-dimers > 1000 ng/mL

D-dimers ≤ 1000 ng/mL

D-dimer ≤ 1000 ng/mL D-dimer > 1000ng/mL
